# Assessment of noise intensity in a dental teaching clinic

**DOI:** 10.1038/bdjopen.2017.10

**Published:** 2017-06-09

**Authors:** Kelly Ferreira da Cunha, Rubem Beraldo dos Santos, Celso Afonso Klien

**Affiliations:** 1 School of Dentistry, Lutheran University of Brazil, Cachoeira do Sul, RS, Brazil

## Abstract

**Objectives/Aims:**

The aim of this study is to evaluate if the duration of the consultation has influence on the intensity of noise in the dental clinic environment.

**Materials and Methods:**

The measurement was performed using the decibelmetre IDETEC 300. It was turned on among 10 dental equipment at basal time (BT) and in the first, second and third hours of activity by 10 times with 1-week interval.

**Results:**

The average noise was 67.39±1.11 dB for BT and 82.38±3.85, 80.99±4.78, and 70.06±6.95 dB for hours 1 to 3, respectively, representing a significant difference (ANOVA, F(3.36)=29.28, *P*<0.0001).

**Conclusion:**

In the first and second hours of clinical care there was more noise intensity in the work environment compared to BT and to the third time. Furthermore, the noise peaks became closer to the limit of 85 dB, which can threaten hearing loss with repeated exposure throughout a dentist’s career.

**Discussion:**

The findings presented here show how much the dental surgeon is exposed to the level of noise since his time of study.

## Introduction

Noise-induced hearing loss (NIHL) can be defined as a permanent sensorineural hearing loss caused by occupational activity. This type of hearing loss is attributed to prolonged exposure to high intensity noise. This loss begins when high frequencies of about 4000 Hz are reached. Once the exposure to the noise is interrupted, hearing loss can be stopped. Normally, the first signs presented by the assessed subject are seen on common tasks such as hearing the ringtone of a phone call. With the progress of the disease, difficulties in the lowest frequencies will arise.^1^ In the literature, it is recorded that several professions have the potential to promote NIHL,^2–5^ including dentistry.

The hearing loss of bigger interest to dental profession is classified as that induced by long exposure to intensive noise. These occupational noises, beyond hearing effects, could have other negative impact to the dentist, such as irritability, nervousness, anxiety, tinnitus, tachycardia, changes in blood pressure, headaches, loss of appetite, stomach pain and insomnia.^6–12^ Of course, the noise in the dental office can also cause discomfort to the patient.^2^

NIHL is an occupational disease that could be considered as the biggest avoidable hearing loss in the world.^7^ Considering the Brazilian legislation, the maximum limit of up to 85 dB to a working time of 8 h daily is allowed in norms 7 and 15 of the Brazilian Work Ministry. In addition, periodic evaluations of hearing are indicated.^8,9^

Dental surgeons are professionals much exposed to the noise of different sources in their offices: background noise, loud music, air conditioners, high-volume aspirators, ultrasonic scalers, high-speed hand pieces, low-speed hand pieces and amalgamators. Only to start the analysis, just the high-speed hand piece can reach a level of noise of 86 dB. Another important factor is that even the arrangement of the furniture in the dental office may increase the noise, so an adequate acoustic project could be useful to improve the well-being of the patients and to prevent NIHL in dentists and auxiliary staff.^10–12^

Some studies have shown that dental surgeons with a level of lower than 85 dB of noise but with 5 or more years of profession can have some grade of hearing loss. It is recommended that these dental surgeons subject themselves to periodic audiologist evaluation and the strict use of equipment of personal protection.^13–17^

According to the presented bibliographical basis, the environment, devices and furniture are factors that can magnify the intensity of the noise in the dental clinic beyond the dental devices.^18^ In addition, the daily work and professional activity time can also justify the study of dentist’s training environment,^12,19^ searching for strategies to stop the risk of hearing loss as early as possible. Therefore, the aim of this study is to evaluate if the clinical consultation time has influence on the intensity of noise in a teaching clinic of dentistry and whether this environment level of noise is in accordance with the maximum level allowed by legislation to reduce the risk of hearing loss.

## Materials and Methods

**Figure 1:**
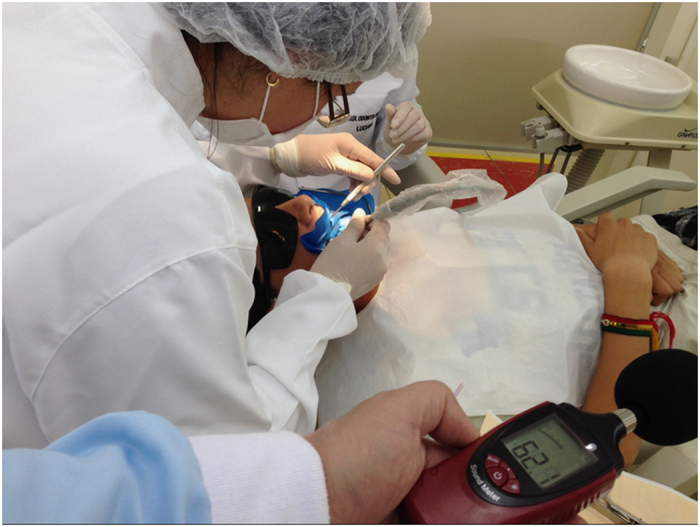
Measurement of noise using the decibelimetre.

The present protocol of research was approved by the Research Department of the Lutheran University of Brazil and received the number 4172015. The method of noise measurement was adapted from the study by Lourenço *et al.*^1^ when the decibelimeter IDETEC 300 (Instrutemp, Belenzinho, SP, Brazil; 30.5–140 dB) was used. The device was placed among 10 dental chairs (a central row of five columns with a pair of chairs each) into the integral clinic. The decibelimetre was placed on the right dental chair in the central column. At each measurement, the device was hold by a single operator, 80 cm far from the chair. The intensity of noise was measured for 5 min at basal time (BT), before starting the clinic, and in the first, second and third hours of activity. These measurements were repeated 10 times with 1-week interval. In each moment, the device was turned on for 5 min and the highest intensity of noise was recorded. A single examiner took note of the results without seeing the earlier records (Figure 1).

At BT, the students arrived and started to prepare their workstation in the clinic. It comprises a dental chair, an auxiliary table and a shelf to put their instruments on; the sources of noise were the talking of the people, their movements and the air conditioning. Students were instructed not to trigger to test the dental equipment during the time of the measurement of environmental noise.

A new measurement was performed at the first hour when all patients were under attention and the students, patients and teachers were in movement into the environment and were sometimes talking also. In addition, with the same background noise of environment as described at BT, several devices working at the same time were observed (hand pieces of high-speed turbine and low-speed, high-velocity suction, ultrasonic instruments, cleaners, vibrators, eventually music and other dental devices). The same data collection method was applied in the second and third hours when each class was finishing.

The obtained data were assessed by means of descriptive statistics and to compare the mean of level noise in all moments by one-way analysis of variance (ANOVA) with Tukey’s *post hoc* test (*P*<0.05 was considered statistically significant).

## Results

At BT, the device was turned on for 5 min and the maximum measured noise of 67.39±1.11 dB was recorded. In the first, second and third hours, 82.38±3.85, 80.99±4.78 and 70.06±6.95 dB, respectively, were recorded. Therefore, there was a statistically significant difference among several times of the assay (ANOVA, *P*<0.001).

**Figure 2:**
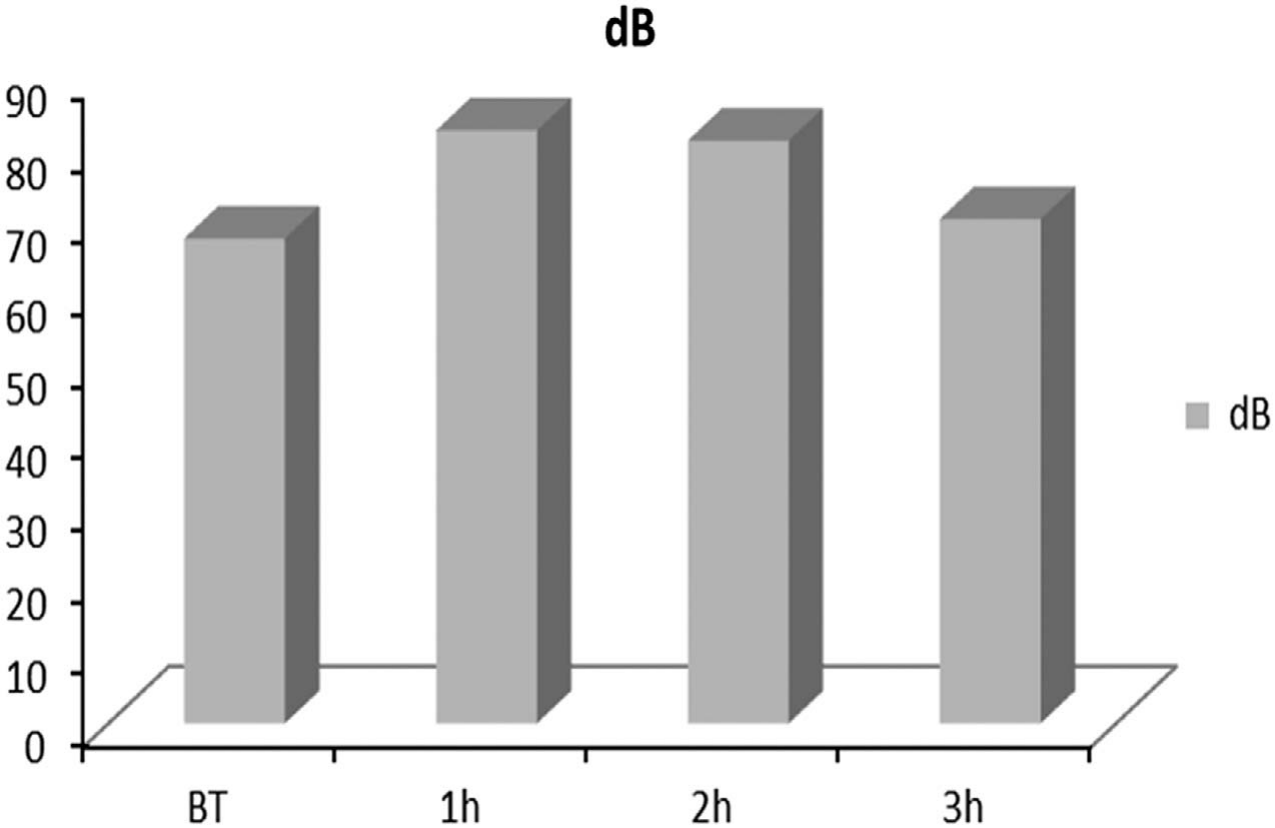
Representation of the average noise intensity in four analysis moments.

In Figure 2, it is possible to observe that the intensity of noise at BT was similar to the third hour of work in the dental clinic, because the numerical difference was not supported statistically. On the contrary, for both these moments, the noise level was statistically lower compared to the first and second hours of activity. Finally, there was no observed difference between the intermediary measurements or between the first one and the last one.

In Table 1, the difference among several measurements was statistically significant (*P*<0.0001).

## Discussion

The noise level observed in this study was obtained using a decibelimetre turned on among 10 workstations in the clinical school of dentistry, where the maximum noise level almost reached the limit allowed by Brazilian law of 85 dB in 8 h of work to prevent NIHL. Even if the highest level has not been continuous and maintained throughout the analysis time, these findings again justify this type of research.

In a study performed at the Dental School of Prince of Songkla University,^18^ the level of noise measured was lower than that level observed in the present study. Whereas the mean in the former study was from 58.1 to 66.43 dB, here the values can reach 82.38 and 80.99 dB in the first and second hours of evaluation, respectively. This difference could be attributed to the methods. In the study of Songkla University, the assessed subjects wore a collar with a decibelimetre attached to determine the personal noise dose in the hearing zone instead of the noise level in working area. The second way was realised as they recorded the noise in each minute obtaining a mean period in contrast to the present study where only the peak of noise in 5 min was recorded. In addition, in that study, the environment was assessed and the level of noise was slightly bigger than those recorded in the hearing zone. These findings in both studies can suggest that the level of noise that really damaged the hearing of students could be a little bit lower than that registered in the dental clinic.

In another study performed at the Oxford Hospital and Oxford School of Dentistry,Bangalore, Karntaka,^12^ the level of noise of the devices for teaching dentistry was measured by a microphone placed near the operator’s ear in a distance of 6 inches from the main noise’s source to simulate the auditive position. The minimal intensity and the maximal intensity of noise were assessed for 30 s with the dental devices triggered only during the cutting moments, when the intensity of noise ranged from 64 to 97 dB. Although those methods are different from those of the present study, the findings are similar, showing that in some moments the arising noise is so high, which makes the environment of learning dentistry with loud frequencies of noise determine the loss of hearing with time, psychological stress and physical stress.^6,11,12,15^

Similar to the present study, an evaluation was carried out in private practices and in the public service from Jundiaí, SP, Brazil.^1^ The measurements were made by a decibelimetre triggered for 5 min. At first, it assessed the environment noise to determine the BT level of noise. After that, the high-speed hand pieces of three different trademarks were used in a distance of 20 cm by different professionals in their dental offices. It was observed that the level of noise at BT was lower in all measurement times, not exceeding the mean of 67.1 dB, whereas when the high speed was triggered the mean of 83.1 dB was reached. This result was not different from the present study, where the noise level was 67.39 dB at BT and 82.38 and 80.99 dB at the first and second hours, respectively. Although these levels of noise never exceeded 85 dB, they increased from the first hour to the second hour during which the noise became higher with the use of dental devices.^1,6,10–12,15,18^

Aside from the devices, it is important to assess the dental environment as it also has an important influence on the intensity of noise.^10–12^ In the present study, the measurements were carried out in the clinical school of dentistry, where it was not possible to say that there is an ideal acoustic control. The hall measured 757.4 m^2^ in area and 2.90 m in height; therefore, the whole volume was 2196.46 m^3^ and the hall contained 73 dental chairs. However, only 10 workstations were used by the students at the same time, although the environment was open and huge and with the dissipation of noise the measurements reached a level near 85 dB. These findings suggest that it would be necessary for a single-private practice or for a dental clinic with more than one workstation to have an adequate acoustic project with the control of shaking and shock and disposition of furniture, observing a minimum size of the clinic hall and contributing to the work process as quiet as possible.^6,11^

The findings presented here show how much the dental surgeon is exposed to the level of noise since his time of study. It is necessary to pay special attention to programs of prevention of hearing loss with the aim of keeping not only the hearing health but also the mental and physical health of those professionals. Considering the facts cited above, the wear of individual ear protection equipment, an adequate maintenance of the dental devices, an acoustic project in the dental clinic hall, and a regular audiometric examination are strongly recommended.^11,12,15^ Also, in the future, it would be necessary to compare the level of noise of private practices with environment of teaching of dentistry, preclinical laboratories and longitudinal audiometric assessments of student’s hearing ability from the first year to the last year of dental school to better understand the risk and to prevent hearing loss throughout the exposure.

## Conclusion

Considering the methods and the data obtained and discussed in this experiment the authors concluded that in the first and second hours of clinical care there is more noise intensity in the work environment compared to BT and the third time. Furthermore, the noise peaks become closer to the limit of 85 dB, which can determine reduction of hearing to the dental surgeon with daily work time and longer time of profession. The findings presented here show how much the dentist is exposed to high intensity of noise since the begging of dental school. So, programs preventing hearing loss with the aim of keeping not only the hearing, but also the mental and physical health of those professionals are extremely necessary.

## Figures and Tables

**Table 1:** Comparison among four samples on all weeks of noise measurement, Cachoeira do Sul, 2015

	*BT*	*1 h*	*2 h*	*3 h*
Week 1	67.67	82.27	81.07	72.53
Week 2	66.84	83.69	82.9	70.01
Week 3	66.44	84.97	79.19	72.85
Week 4	68.95	77.96	83.45	69.56
Week 5	65.53	82.14	80.5	71.54
Week 6	68.12	83.18	78.47	73.44
Week 7	66.75	89.97	84.6	50.89
Week 8	69.12	75.5	77.88	75.67
Week 9	67.01	81.9	90.02	72.11
Week 10	67.5	82.19	71.9	72.02
Mean	67.39	82.38^a^^,^^b^	80.99^a^^,^^b^	70.06
s.d.	1.119	3.857	4.789	6.950

Abbreviations: ANOVA, analysis of variance; BT, basal time.

Values are means±s.d. BT (*n*=10), 1 h after (*n*=10), 2 h after (*n*=10) and 3 h after (*n*=10). Weeks 1 to 10 represent each moment of measurement; statistical analysis by one-way ANOVA followed by Tukey’s *post hoc* test. Symbols represent the comparison between groups by *post hoc* analysis: *P<0.05 compared to BT measurement (one-way ANOVA, F(3.36)=29.28; *P*<0.0001; Tukey’s *post hoc* test).

a *P*<0.05 when 1 and 2 h were compared to BT.

b *P*<0.05 when 1 and 2 h were compared to 3 h after.
